# Expectations About Care for Aging Parents and One’s Own Future Care Among Korean Middle-Aged Adults

**DOI:** 10.1093/geroni/igaf122.2651

**Published:** 2025-12-31

**Authors:** Haeun Yoo, Eunju Lee, Kyungmin Kim

**Affiliations:** Seoul National University, Seoul, Republic of Korea; Seoul National University, Seoul, Republic of Korea; Seoul National University, Seoul, Republic of Korea

## Abstract

Korean adults have traditionally anticipated providing care to their aging parents, while also hoping their own children will reciprocate similar care when needed. However, as family care norms have weakened, people’s expectations about care are in flux—some anticipate family care, some expect formal care (e.g., nursing homes or professional care workers), while others have no clear expectation about care. Further, people may have different expectations between care for aging parents and their own future care. This study examined middle-aged adults’ expectations about (a) care for parents and (b) their own future care (i.e., family care, formal care, or uncertain) and how combinations of these care expectations were related to aging anxiety. Using data from 2014 Korean Baby Boomer Panel Study, we analyzed a sample of middle-aged adults (N = 1,748; aged 51–59), who have at least one living parent and one living child. We found that 36% expected family care for parents but formal care for themselves, followed by expecting formal care for both (32%). Expecting formal care for parents but family care for oneself was least common (1%), while expecting family care for both was also rare (6%). Additionally, 24% indicated uncertain expectations about either or both. Regression analyses revealed that individuals who expected formal care for both or who had no clear expectations experienced greater aging anxiety than those expecting family care for both. These findings highlight the importance of planning for aging parents’ and own future care needs in the context of changing care norms.

